# *Teladorsagia circumcincta* beta tubulin: the presence of the E198L polymorphism on its own is associated with benzimidazole resistance

**DOI:** 10.1186/s13071-020-04320-x

**Published:** 2020-09-07

**Authors:** María Martínez-Valladares, Elora Valderas-García, Javier Gandasegui, Philip Skuce, Alison Morrison, Verónica Castilla Gómez de Agüero, Maria Cambra-Pellejà, Rafael Balaña-Fouce, Francisco A. Rojo-Vázquez

**Affiliations:** 1 Instituto de Ganadería de Montaña (CSIC-Universidad de León), Grulleros, 24346 León, Spain; 2 Departamento de Sanidad Animal, Facultad de Veterinaria, Universidad de León, Campus de Vegazana, 24071 León, Spain; 3 Departmento de Ciencias Biomédicas, Facultad de Veterinaria, Universidad de León, 24071 León, Spain; 4 Instituto de Salud Global de Barcelona (ISGlobal), Barcelona, Spain; 5 Moredun Research Institute, Pentlands Science Park, Edinburgh, EH26 0PZ UK

**Keywords:** Benzimidazole resistance, *Teladorsagia circumcincta*, FECRT, EHT, β-tubulin gene, Polymorphism

## Abstract

**Background:**

Benzimidazole resistance is associated with isotype-1 β-tubulin gene F200Y, E198A and F167Y SNPs. In this study, the recently described polymorphism E198L was reported and analysed in *Teladorsagia circumcincta*.

**Methods:**

The benzimidazole phenotypic resistance was measured by the faecal egg count reduction test (FECRT) and the egg hatch test (EHT) using a discriminating dose (DD) in 39 sheep flocks. Around 1000 larvae collected before and after treatment were used for DNA extraction. The resistant species identified in all flocks was *T. circumcincta*. The resistance alleles frequencies were measured for F200Y and E198A. A 371-bp fragment of the isotype-1 β-tubulin gene was analysed, including the three codons of interest, and a new pyrosequencing assay was designed for testing E198L.

**Results:**

The percentage of resistant flocks was 35% by FECRT or 26% by EHT; however, F200Y and E198A SNPs were absent in *T. circumcincta*. The amplification of a 371-bp fragment confirmed the absence of F167Y and F200Y in 6 resistant flocks. Regarding codon 198, all samples after treatment carried a leucine (CTA). A pyrosequencing assay analysed the allele frequencies for the first two bases at codon 198 independently, G/C and A/T. The correlation between C and T frequencies was almost 1 (*r* = 0.929, *P* < 0.0001) and the mean value of both was calculated to measure the leucine frequency; this value ranged between 10.4–80.7% before treatment, and 82.3–92.8% after treatment. High and similar correlations were reported between the genotypic variables (C frequency, T frequency or mean of both frequencies) and phenotypic resistance (*r* > 0.720, *P* < 0.0001), although negatively associated with the FECRT and positively with the EHT. According to multivariate linear regression analysis, the T frequency was the most significant variable influencing the phenotypic resistance (FECRT or EHT; *P* < 0.0001). In the EHT, 67.1% of the phenotypic variability is associated with the T frequency but in the FECRT only 33.4%; therefore, the EHT using a DD seems to detect the genotypic resistance more accurately than the FECRT.

**Conclusions:**

The E198L polymorphism can confer BZ resistance on its own in *T. circumcincta*. 
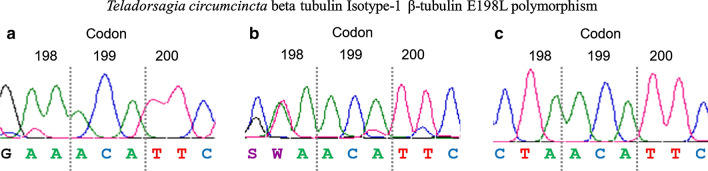

## Background

Infections by gastrointestinal nematodes (GIN) in grazing ruminants are highly prevalent worldwide. The importance of GIN infection is due to the economic losses associated with reduced weight gain, milk yield and wool production [1]. Since the first anthelmintic was introduced to the market in the 1940s, these drugs have been the most effective way to control GIN in grazing animals, but this approach is now under threat since the emergence of anthelmintic resistance (AR). The efficacy of anthelmintics is being reduced by the overuse and misuse of these drugs, especially those belonging to benzimidazole (BZ) family, the first anthelmintics to be commercialized, mainly because they are highly effective against GIN and have a low cost per dose.

Nowadays, AR has been reported in many GIN species to BZs in many countries and multiple host animals [2], but also to other drugs such as macrocyclic lactones and even the most recently commercialized anthelmintics, such as monepantel [3] and derquantel [4]. The presence of multi-drug resistance to different anthelmintic families has been described in different countries and GIN species [5–7]. Among ruminants, sheep are especially affected by the presence of AR, often being parasitized by *Teladorsagia circumcincta*, *Trichostrongylus* spp., *Haemonchus contortus*, the most prevalent and resistant species [3, 5–7].

Since the number of anthelmintics is quite limited, and in the short to medium term it is unlikely that new drugs will appear on the market, a more sustainable use of anthelmintics is required. For that, AR has to be detected as early as possible to avoid its spread. The *in vivo* faecal egg count reduction test (FECRT) has been extensively used for anthelmintic efficacy testing at farm level due to its simplicity and can be used for testing all anthelmintic families; however, it is time-consuming and expensive because it involves two separate farm visits. With the aim of avoiding these drawbacks, the *in vitro* egg hatch test (EHT) was developed for the detection of BZ resistance; this test is based on the fact that eggs from resistant isolates embryonate and hatch at higher drug concentrations than do those from susceptible isolates. Although the EHT was developed and standardized using serial dilutions of thiabendazole (TBZ) to calculate the dose required to prevent 50% of the viable eggs from hatching (ED_50_) [8], a simplified form of the test can be performed using a discriminating dose (DD) [9].

Rapid and accurate molecular tests are not available for most drug classes and helminth species, however, a DNA-based test for detection of BZ resistance in GIN is available. In the latter, resistance to BZ is associated with a single nucleotide polymorphism (SNP) at specific codons within the gene coding for isotype-1 β-tubulin [10]. The substitution of a phenylalanine (TTC) for a tyrosine (TAC) at codon 200 (so-called, F200Y) has been associated with BZ resistance in several different GIN species (e.g. *H. contortus*, *T. colubriformis*, *T.circumcincta*, *Cooperia oncophora* and *Ostertagia ostertagi*). Less frequently, other SNPs have been associated with BZ resistance, either in combination with others or on their own; for example, the same SNP as previously described was found at codon 167 (F167Y) and a point mutation of glutamic acid (GAA) to alanine (GCA) at codon 198 (E198A). Ramünke et al. [11] used a pyrosequencing assay to measure the BZ resistance-associated SNP frequencies in *T. circumcincta*, *Trichostrongylus* spp. and *H. contortus*. Although the three SNPs seemed to be very widely distributed across the world, in our previous studies, we were unable to detect any of these in BZ resistant *T. circumcincta* larvae from Spain [12, 13]. On this basis, in the present study, a recently described polymorphism at codon 198 was analyzed in *T. circumcincta* larvae, a substitution of glutamic acid (E) by leucine (L). The association between the frequency of this polymorphism and the phenotypic resistance measured by FECRT and EHT was studied.

## Methods

### Selection of flocks

The study was conducted during the years 2015 and 2016 in the northeast of Spain with the collaboration of the livestock cooperative COBADU (Cooperativa Bajo Duero). The flocks had to be without any anthelmintic treatment 3 months before their selection and under grazing conditions. The first step was the identification of those farms infected by GIN by means of a faecal egg count (FEC). For this purpose, individual faecal samples from the rectum of 20 animals were randomly taken in each flock. Faecal samples were analysed individually by a modified McMaster technique and using a saturated solution of sodium chloride (density = 1.2 g/ml) for egg counting. The sensitivity of this technique was 15 eggs per gram (epg). In total, 39 flocks naturally infected by GIN were included in the study.

### Faecal egg count reduction test (FECRT)

FECRT was carried out in flocks with a mean FEC higher than 100 epg, which proved to be 34 from the original 39 total. In each flock, 10 animals were randomly selected to be treated orally with netobimin (Hapasil^®^), a BZ drug (an albendazole pro-drug), at the recommended dose (7.5 mg/kg bodyweight). Each animal was weighed before treatment in order to adjust the individual dose. Rectal faecal samples were taken from every sheep on the day of treatment (day 0) and 10–14 days post-treatment. All samples were processed and analysed individually as described above (“Selection of flocks” section) the following day after sampling. The reduction in the number of eggs was calculated using the following formula:

$${\text{FECRT }}\left( \% \right)\, = \, 100\, \times \,( 1\, - \,\left( {{\text{Mean FEC day 1}}0\,{-}\, 1 4/{\text{Mean FEC day }}0} \right))$$

### Egg hatch test

EHT was performed in all flocks at a discriminating dose (DD) of 0.1 μg/ml of TBZ (Sigma-Aldrich, Madrid, Spain) as previously described by Coles et al. [14]. A stock solution of TBZ was prepared by dissolving the commercial compound in dimethylsulfoxide (DMSO) (Sigma-Aldrich). The final concentration was reached by adding 10 µl of the stock TBZ solution into 1.99 ml of a suspension containing approximately 100 GIN eggs/ml in purified water. All assays were done in triplicate. Eggs were incubated with the drug for 48 h at 23 °C in 24 multi-well plates; 3 control wells with 10 µl of DMSO were also included in each assay.

The hatching ratio (Hdd) was used as the BZ resistance indicator in each EHT, it refers to the percentage of eggs hatching at DD, corrected by hatching in control wells. Given that when the ED_50 _≥ 0.1 μg/ml is indicative of resistance [14], a value of Hdd ≥ 50% at 0.1 μg/ml was considered as indicative of resistance [9].

### Species identification

A pool of faecal samples was collected before and after treatment for a later egg concentration. Eggs were then incubated at 23 °C overnight for collection of first stage larvae (L1). A total of approximately 1000 L1 from each flock and sampled day was stored in 70% ethanol until DNA extraction was performed. DNA was extracted using the Speed Tools Tissue DNA Extraction Kit (Biotools, Madrid, Spain) as per the manufacturer’s instructions.

Species identification was performed on pooled L1 extracts using the ovine nematode panel on an AusDiagnostics Multiplexed Tandem PCR (MT-PCR) machine (AusDiagnostics Pty. Ltd., Beaconsfield, Australia) at the Moredun Research Institute, exactly as outlined in Roeber et al. [15].

### Determination of the resistance allele frequencies for E198A and F200Y

DNA extracted from the ~ 1000 L1 samples was used for the determination of allele frequencies (“Species identification” section).

The determination of allele frequencies at SNPs 198 and 200 of the gene encoding isotype-1 β-tubulin in *Teladorsagia spp.* was carried out following the protocol and primers previously described by Esteban-Ballesteros et al. [13]. The allele frequencies were determined in triplicate for each sample and the arithmetic mean was calculated. The frequencies of the resistant allele with values equal to or lower than 10% were considered as technical background and, therefore, were designated as 0% in the data analysis.

### Sequence analysis of the isotype-1β-tubulin gene

A 371-bp fragment of the *T. circumcincta* isotype-1 β-tubulin gene was amplified, including the codons 167, 198 and 200. The sequence analysis was performed in pooled L1 obtained from faecal samples collected before and after treatment in 6 different flocks and in 40 individual L1 obtained from faecal samples collected before treatment in 2 resistant flocks.

For this, a pair of primers (forward: 5′-GTT CGG GTA TGG GCA CTT T-3′; reverse:5′-TGA GAT CGC CAT AAG TTG GA-3′), was designed based on the mRNA sequence, GenBank accession number Z69258. PCR cycling conditions were 95 °C for 10 min followed by 40 cycles of 95 °C for 45 s, 60 °C for 30 s and 72 °C for 45 s followed by 10 min at 72 °C and 4 °C to finish; DNA mplitools HotSplit Master Mix 2 × (Biotools) was used for the amplification. Specific PCR products were run on a 1.5% agarose gel stained with Gel Red (Biotium, Hayward, USA) and bands were purified with the Speed Tools PCR Clean-up Kit (Biotools) for later sequencing at the Laboratory of Instrumental Techniques (University of León, Spain).

In the same way, this PCR was performed with DNA from pooled L1 collected from 16 flocks after treatment. All these PCR products were mixed together as a single sample. This sample was cloned into a p-GEMT Easy vector (Promega, Madrid, Spain) and transformed into *Escherichia coli* JM109 competent cells (Promega, Madrid, Spain). Eleven clones were sequenced afterwards using universal primers SP6 and T7.

Alignment and analysis of all partial sequences were carried out with the DNASTAR software program (version 14.1).

### Determination of the resistance allele frequencies for E198L (GAA > CTA)

Initially, a new pair of PCR primers was designed to amplify a138-bp fragment of the *T. circumcincta*isotype-1 β-tubulin gene including the codon 198 (forward: 5′-TGG AAC CTT ACA ATG CCA CTC-3′; reverse: 5′-TGA GAT CGC CAT AAG TTG GA-3′). The forward primer was labelled with biotin at the 5′-end. PCR cycling conditions were 95 °C for 10 min followed by 40 cycles of 95 °C for 30 s, 60 °C for 30 s and 72 °C for 30 s followed by 10 min at 72 °C and 4 °C to finish; DNA Amplitools HotSplit Master Mix 2 × (Biotools, Spain) was used for the amplification in a 50 μl reaction volume. PCR products were run on a 1.5% agarose gel. Prior to pyrosequencing, a 7 μl aliquot of each PCR product was tested by agarose gel electrophoresis. The specificity of the primers was confirmed initially by running the same PCR but with DNA extracted from *T. colubriformis* and *H. contortus* adults.

Pyrosequencing of the resulting PCR fragments was done using the sequencing primer (5′-ATT ATC GAT RCA GAA YGT T-3′). The pyrosequencing assay was carried out using a DNA PSQTM96 MAsystem (Biotage, Uppsala, Sweden) according to the manufacturer’s recommendations. For this, 40 μl of PCR product was added to 37 μl 2× Binding buffer (Biotage) plus 3 μl streptavidin sepharose beads (Sigma, Madrid, Spain) in a 96-well plate and then agitated for 5 min at room temperature to allow binding of biotin-labelled DNA to the beads. The beads were processed using the sample preparation tool and reagents (Biotage) then dispensed into the assay plate with 40 μl of 0.4 μM sequencing primer per well. Positive controls (identified in “Sequence analysis of the isotype-1β-tubulin Gene” section) representing susceptible and resistant samples were included in each assay. The analysis of the bases was performed independently from each other.

The pyrosequencing assay was performed at least 3 times for each sample and the arithmetic mean was calculated. The frequencies of the resistant allele with values equal to or lower than 10% were considered as technical background and, therefore, not classed as resistant.

The G/C and A/T frequencies were calculated for each sample. The mean frequency of C and T was then calculated as a measurement of the frequency of leucine at this codon.

### Statistical analysis

The FECRT, along with the lower limit for a 95% confidence interval was calculated according to the recommendations of WAAVP [13]. When the FECRT value is less than 95% and the lower confidence limit less than 90%, the flock should be considered resistant to a given anthelmintic. If only one criterion is described then the flock must be classified as suspicious of resistance but when none criterion is accomplished, the flock is classified as susceptible.

The data were then analysed using the Statistical Computer Package for Social Science (SPSS). The Kolmogorov-Smirnov test was carried out to determine if data were normally distributed. The statistical relationship between the different variables (FECRT, EHT, allele frequencies and species identification) was calculated using the non-parametric Spearman’s rank correlation test. Multivariate linear regression analyses were performed to assess any associations between the dependent variables (FECRT and EHT) and the independent variables (allele frequencies, species identification). A forward stepwise selection procedure was used to select the variables that were significantly associated (*P* < 0.10) with the different independent variables tested.

## Results

### Phenotypic characterization of anthelmintic resistance

A total of 39 flocks were included in the study. The FECRT was performed on 34 of them; the mean FEC before treatment was 188 epg. According to the FECRT, the treatment was effective in 21/34 flocks (62%), with a FECRT higher than 95%. In one of the remaining flocks (3%), the FECRT was higher than 95% although the lower confidence limit was less than 90%, suggesting suspicious of resistance, and the rest (12/34) were declared as resistant with a FECRT lower than 90% (35%). It is noteworthy that in 2 flocks (flocks 23 and 35), the FEC was even higher after the treatment (Table 1, Additional file 1: Table S1, Additional file 2: Table S2).

**Table 1:** Anthelmintic resistance level for each flock according to the FECRT, including the lower confident limit (CI), EHT and percentage of the allele frequency before and after treatment

Flock ID	FECRT	Lower CI	EHT (Hdd)	Mean % C and % T	
				Before treatment	After treatment
1	100.0	100	11.3	10.4	–
2	100.0	100	2.5	8.0	–
3	100.0	100	4.0	8.7	–
4	100.0	100	6.2	18.1	–
5	100.0	100	1.5	12.7	–
6	100.0	100	4.0	5.8	–
7	100.0	100	1.7	24.2	–
8	100.0	100	18.9	12.0	–
9	–	–	91.6	65.8	–
10	98.6	96	32.9	24.8	–
11	–	–	22.4	12.0	–
12	–	–	17.1	10.1	–
13	100.0	100	24.3	6.8	–
14	100.0	100	33.3	11.9	–
15	–	–	19.6	8.2	–
16	96.8	92	30.9	11.9	–
17	97.1	89	23.5	11.1	–
18	–	–	12.2	13.4	–
19	100.0	100	23.5	8.2	–
20	96.1	92	43.7	10.5	–
21	100.0	100	48.3	8.6	–
22	34.0	0	76.1	80.7	86.7
23	− 110.0	0	44.9	60.9	85.8
24	58.5	27	59.3	75.6	86.0
25	100.0	100	43.6	34.5	90.2
26	17.0	0	72.3	42.8	92.5
27	60.0	25	71.3	71.1	–
28	87.9	73	34.5	45.8	84.8
29	7.2	0	93.4	80.3	86.2
30	69.4	41	41.0	57.1	89.5
31	97.9	92	42.6	45.3	92.8
32	42.1	0	54.4	60.5	91.2
33	88.1	69	40.3	65.3	88.7
34	91.3	75	61.6	78.3	91.1
35	− 100.0	0	89.6	78.4	–
36	100.0	100	35.8	52.7	88.5
37	100.0	100	40.2	50.1	90.8
38	97.5	90	71.7	58.9	89.3
39	100.0	100	19.7	31.3	82.3

The level of resistance using the EHT was measured in all flocks. Hdd was lower than 50% in 29/39 flocks (74%), susceptible to ABZ, and consequently 26% of them declared as resistant. Among the resistant flocks (10/39), the Hdd ranged from 54.4% to 91.6% (mean: 72%) (Table 1, Additional file 2: Table S2).

**Fig. 1:**
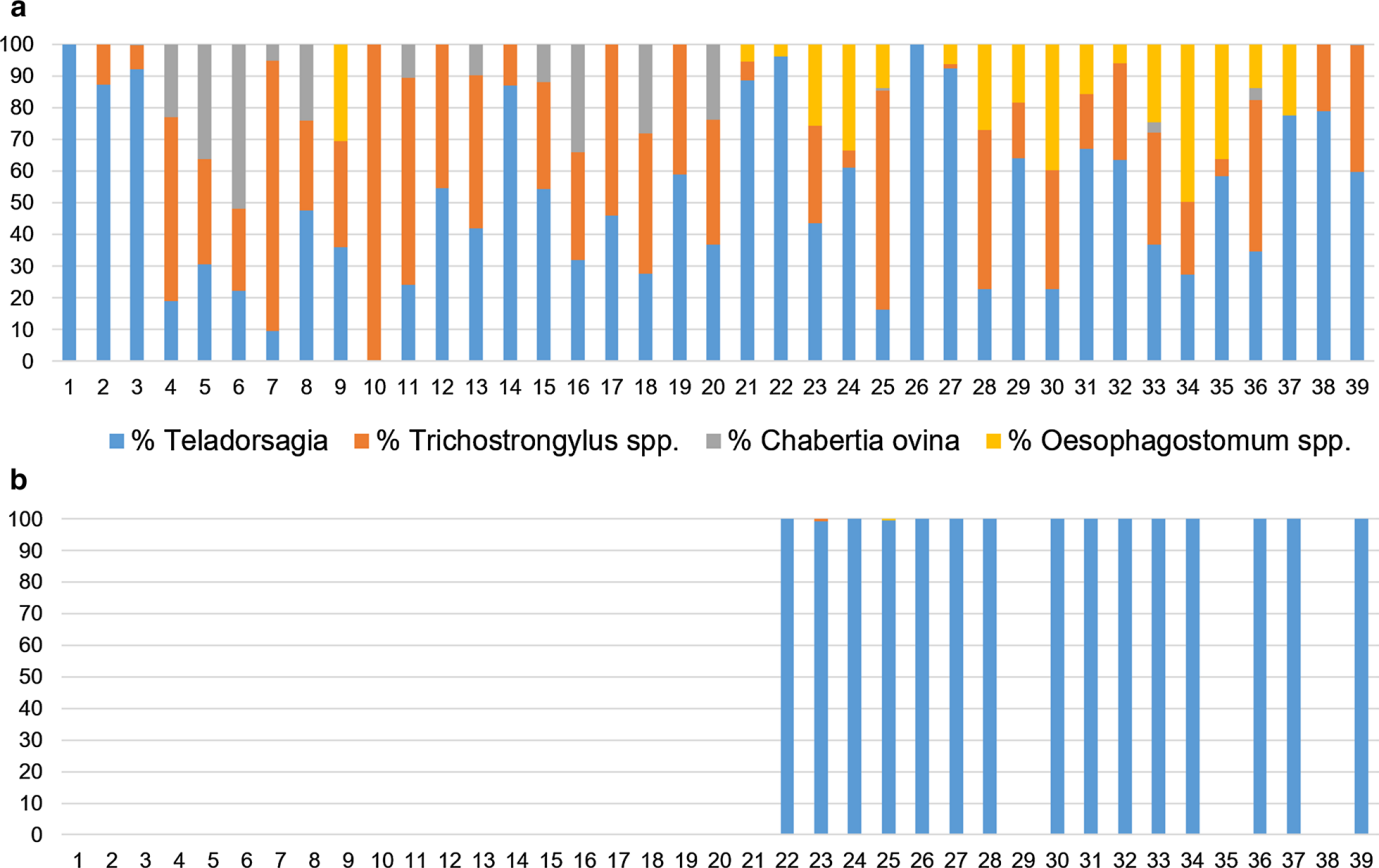
Percentage of gastrointestinal nematode species detected in each flock before (**a**) and after (**b**) treatment. Y axis represents the percentage of each specie and the X axis the flock identification. As shown in the legend, the blue colour indicates the percentage of *T. circumcincta*; the orange, *Trichostrongylus* spp.; the grey, *Chabertia ovina*; and the yellow, *Oesophagostomum* spp

For GIN species identification, the MT-PCR was carried out on all flocks before treatment. The most prevalent species before treatment were *T. circumcincta*, present in 38 flocks with values between 9.5–100% (mean: 53.1%), and *T. colubriformis*, found in 35 flocks with values between 1.3–100% (mean: 35.5%). *Oesophagostomum* spp. and *C. ovina* were present in 17 and 16 flocks, with values between 3.9–49.6% (mean: 21.9%) and 0.4–51.9% (mean: 16.7%), respectively. *Haemonchus contortus* was not found in any flock. After treatment, MT-PCR was performed in 15 flocks; in 13 flocks out of 15, all larvae were identified as *T. circumcincta* (100%). In the other two flocks, *T. circumcincta* represented 99.2 and 99.6% of all larvae, and the remaining percentages were 0.8% *T. colubriformis* and 0.4% *Oesophagostomum* spp., respectively. Figure 1 shows the percentage of the different species of GIN in each flock, before (Fig. 1a) and after (Fig. 1b) treatment. As can be observed, the resistant species resulted in all flocks sampled was *T. circumcincta* (Additional file 2: Table S2, Additional file 3: Table S3).

### Genotypic characterization of anthelmintic resistance

Due to the fact that the main resistant species was *T. circumcincta* all genotypic characterization was performed in this species. Initially the allele frequencies for E198A and F200Y were measured by pyrosequencing. None of the resistant alleles previously shown to be related with BZ resistance was found before treatment in any codon. After treatment, only one out of the 16 flocks analysed showed a frequency of the resistant allele higher than 10% at codon 200 (11.6 ± 4.8%); this flock was classed as highly resistant, with a value of 7% after the FECRT and 93.4% for the Hdd. The resistant allele at codon 198 was not found in any flock (data not shown).

With the aim to confirm the previous results, a 371-bp fragment of the *T. circumcincta* β-tubulin gene was amplified, with a homology of almost 100% of isotype-1 β-tubulin gene, and including the three codons of interest. The amplification of this fragment confirmed the absence of SNPs F167Y and F200Y before and after treatment in DNA samples from pooled L1 collected from 6 resistant flocks.

**Fig. 2:**
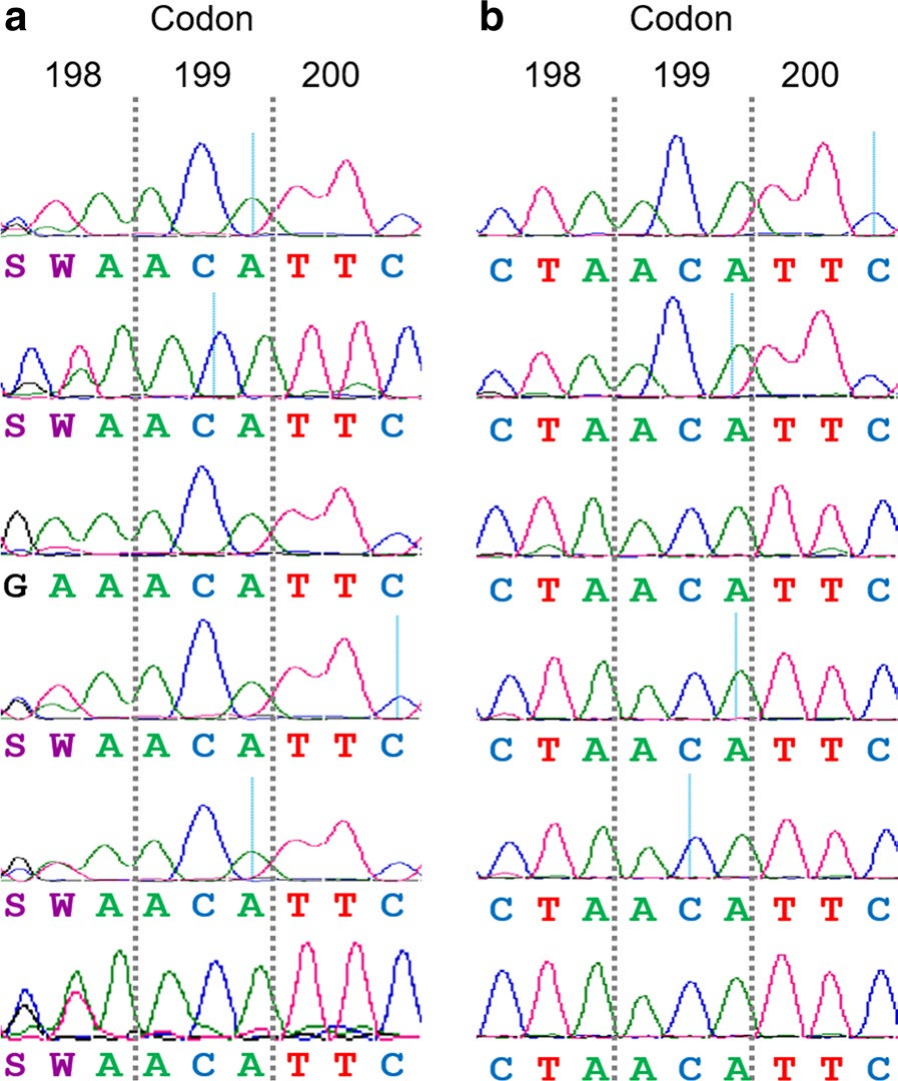
Sanger sequencing results of the three codons 198, 199 and 200 of the beta tubulin gen of *T. circumcincta* obtained from 6 resistant flocks sampled before (**a**) and after (**b**) treatment. The order of the flocks in the figure is maintained from the top to the bottom in the two columns-before and after treatment

Regarding codon 198, in the samples collected before treatment, the amino acid present was not clear, with multiple peaks on chromatograms; the possibility of two nucleotides in the first two positions of the codon (G/C A/T A) could lead to four different amino acids (glutamic acid, glutamine, valine or leucine) (Fig. 2a). However, the 6 resistant samples collected after treatment showed a leucine (CTA) at this position (Fig. 2b). Moreover, 11 clones obtained after cloning pooled PCR products from all resistant flocks after treatment were sequenced; all of them showed the homozygous genotype for leucine (CTA/CTA).

**Fig. 3:**
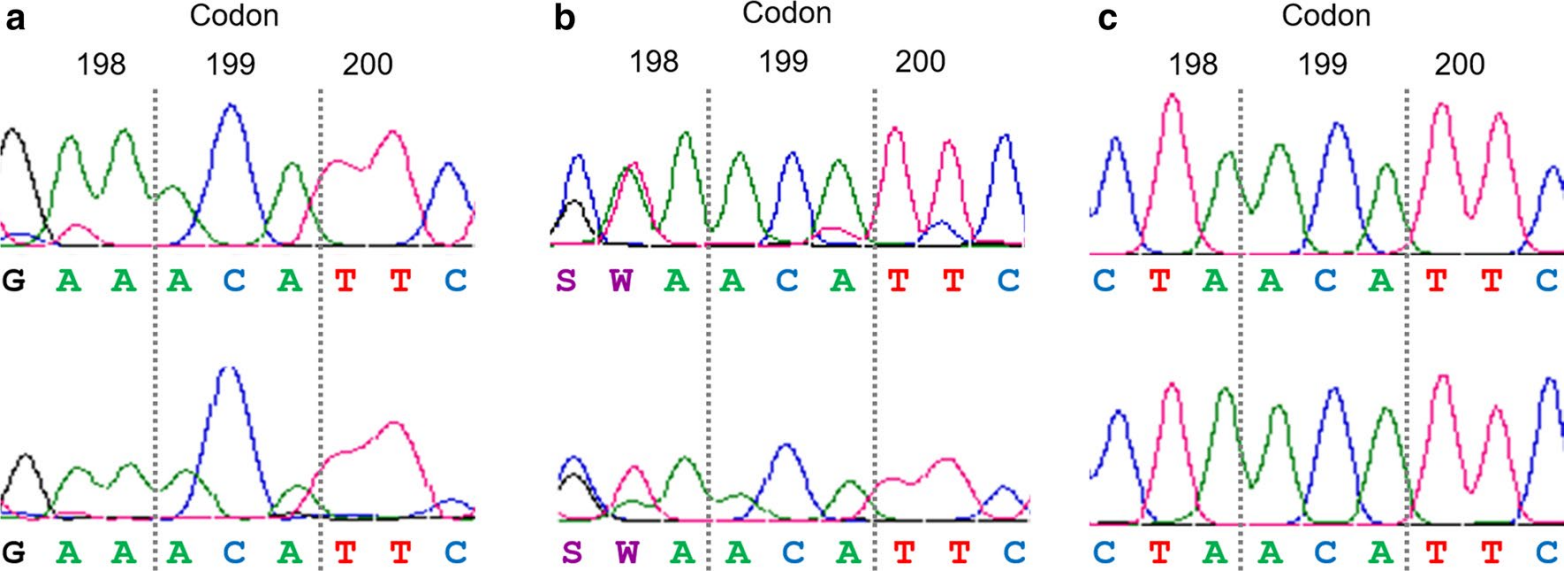
Sanger sequencing results of the three genotypes obtained after sequencing individual L1 larvae of *T. circumcincta*. **a** Susceptible homozygous genotype for glutamic acid. **b** Susceptible-resistant heterozygous. **c** Resistant homozygous genotype for leucine

With the aim to clarify the genotype before treatment, 40 individual L1 collected before treatment from two resistant flocks, were analysed after the amplification of the 371-bp fragment. As a result, 36 out of 40 L1 showed the homozygous genotype for glutamic acid (GAA/GAA) (GenBank: MT818234) (Fig. 3a), another two were heterozygous at codon 198 (GAA/CTA) (GenBank: MT818235) (Fig. 3b) and the remaining two were homozygous for leucine (CTA/CTA) (GenBank: MT818236) (Fig. 3c).

A pyrosequencing assay was performed according to the previous results to analyse the allele frequencies for the first two bases at codon 198 independently, G/C and A/T. In the samples analysed after treatment the C and T frequencies were high and very similar to each other, coding for leucine. The mean value of both frequencies was calculated to measure the frequency of this polymorphism and ranged from 82.3 to 92.8%. Regarding the 39 samples before treatment, 35 showed similar frequencies of G and A, or C and T, coding for glutamic acid or leucine. In these 35 flocks, the frequency of leucine, measured as the mean value between C and T, ranged from 10.4 to 80.7%. There were four flocks, however, with similar frequencies among the 4 bases (flocks 28, 31, 36 and 37), ranging from 39.0 to 59.8%; so, the frequencies were closer between G and T, or C and A. Therefore, the coding amino acid was not well defined in these four flocks before treatment although after treatment all of them showed a high frequency for leucine (84.8–92.8%) (Additional file 2: Table S2, Additional file 3: Table S3).

**Fig. 4:**
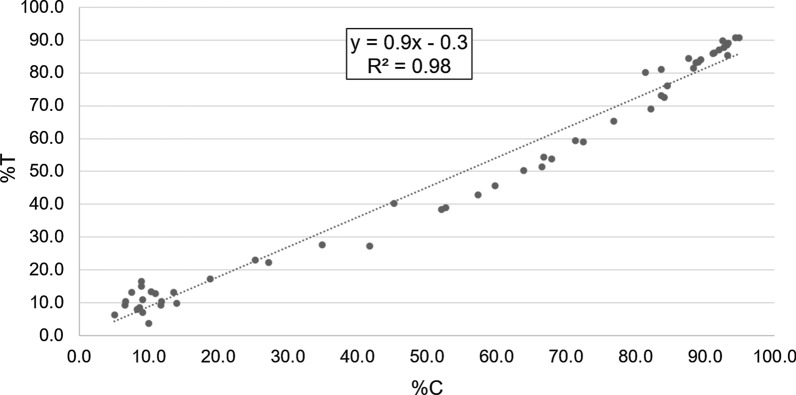
Plot graph and linear regression parameters between the frequency of each nucleotide (C and T) in the codon 198 of *T. circumcincta*, obtained by pyrosequencing

Nevertheless, when the linear regression model between C and T frequencies was developed for each sample, results strongly agreed with the model (Fig. 4). Besides, the correlation between the two frequencies was almost 1 (*r* = 0.929; *P *< 0.0001) (Table 2).

**Table 2:** Correlation matrix between the FECRT values, EHT values, C frequency, T frequency, mean frequency (C and T), percentage of *T. circumcincta* per flock, percentage of *T. circumcincta* carrying the leucine (LEU) amino acid at codon 198

	FECRT	EHT	% C	% T	Mean (% C and %T)	% *T. circumcincta*	% *T. circumcincta* carrying LEU
FECRT	1	− 0.770**	− 0.764**	− 0.724**	− 0.738**	− 0.130	− 0.638**
*P-*value		< 0.0001	< 0.0001	< 0.0001	< 0.0001	0.465	< 0.0001
EHT		1	0.782**	0.730**	0.743**	0.286	0.755**
*P-*value			< 0.0001	< 0.0001	< 0.0001	0.077	< 0.0001
% C			1	0.929**	0.966**	− 0.005	0.722**
*P-*value				< 0.0001	< 0.0001	0.976	< 0.0001
% T				1	0.984**	0.101	0.794**
*P-*value					< 0.0001	0.542	< 0.0001
Mean (% C and % T)					1	0.037	0.761
*P*-value						0.821	< 0.0001
% *T. circumcincta*						1	0.621**
*P*-value							< 0.0001
% *T. circumcincta* carrying LEU							1

** *P* < 0.0001

### Association between phenotypic and genotypic variables

The association between the two measurements of the phenotypic resistance, FECRT and EHT, was high (*r* = 0.770; *P* < 0.0001). Moreover, both measurements were highly correlated (*r* > 0.720; *P* < 0.0001) with the genotypic variables—C frequency, T frequency and the mean of both frequencies. The higher the frequency of both nucleotides, the lower the efficacy of the anthelmintic (low FECRT and high Hdd) (*P* < 0.001). Despite the fact that neither the FECRT nor the EHT was associated with the percentage of *T. circumcincta*, the percentage of larvae carrying the leucine amino acid (measured as mean frequency of C and T) were highly correlated with both measurements; the higher the percentage of these larvae, the lower the FECRT (*r* = − 0.638; *P* < 0.0001), and the higher the Hdd (*r* = − 0.755; *P* < 0.0001) (Table 2).

For each dependent variable, there were different multivariate linear regressions. Table 3 shows those with the highest coefficient of multiple determinations (*R*^2^). The values of each *R*^2^ mean that 33.4%, 67.1% and 67.0% of variance of each dependent variable (FECRT or EHT) is explained by the independent variables included in each model. The models showed that the phenotypic resistance was influenced by the T frequency at the second position of codon 198, negatively with the FECRT and positively with the Hdd, with a level of significance *P* < 0.01 in all models. Model 3 confirmed that the percentage of *T. circumcincta* carrying the leucine amino acid at codon 198 influenced slightly the Hdd measured by the EHT (*P* = 0.072) (Table 3).

**Table 3:** Multivariate linear regression analysis indicating the *P*-value of the model and B as the regression coefficient with their corresponding *P*-value

Model	Dependant variable	Significant independent variable(s)	*R*^2^	*P*	B	95% CI of B		*P*
						Minimum	Maximum	
1	FECRT	Intercept	0.334	0.000	117.106	90.679	143.533	< 0.0001
		% T			− 1.245	− 1.878	− 0.613	< 0.0001
2	EHT	Intercept	0.671	0.000	8.732	0.383	17.081	0.041
		% T			0.888	0.681	1.096	0.000
3	EHT	Intercept	0.670	0.072	10.195	1.942	18.448	0.017
		% T			0.597	0.220	0.974	0.003
		% Resistant *T. circumcincta*			0.420	− 0.040	0.880	0.072

The strength of association is represented by the coefficient of multiple determination (*R*^2^)

## Discussion

The early detection of AR in GIN infecting ruminants is a crucial measure in curtailing its spread and establishing alternative methods for controlling these infections. Conventional techniques such as the *in vivo* FECRT or *in vitro* tests such as the EHT have been used for decades to detect the presence of resistance against different anthelmintic families since the first guidelines were published [14]. Arguably, quicker and more reliable methods of screening are needed to scale up the resistance detection in preventive campaigns to inform deworming practices.

The EHT has been used as an *in vitro* test for the detection of BZ resistance in previous studies using different TBZ concentrations to calculate the ED_50_ [16–18]; however, in the present study we used a single DD as a simplified form of the test. According to our results, the percentage of resistant flocks in the northwest of Spain during 2015–2016 was 35% by FECRT or 26% by EHT. It is important to note that the prevalence of BZ resistance between 2006 and 2011 in the same area of study was 13.6% measured by FECRT [17]. Consequently, it was seen an increment over the time of more than double in the same area although it is important to note that the same flocks were not sampled in both studies. The EHT demands less time, effort and cost than FECRT. Using a DD of 0.1 μg/ml of TBZ in the EHT seems a good alternative to the *in vivo* method since the levels of resistance measured by both tests were quite similar and comparable in our study, with a highly significant level of correlation (*r* = 0.77; *P* < 0.0001). This finding is in accordance with the study carried out by Rialchet al. [19], who also reported very high correlations between the FECRT and the ED_50_ calculated by the EHT (*r* =  0.88; *P* < 0.01).

That said, the detection of specific mutations related to BZ resistance could result in a more specific and accurate measurement than *in vivo* and *in vitro* methods. The F200Y mutation at isotype-1 β-tubulin gene still appears to be the most important polymorphism associated with BZ resistance in nematodes infecting ruminants, however, it does not adequately explain all BZ resistant phenotypes, other mutations present in the same gene has also been reported, F167Y and E198A [20]. Our research group in Spain tried to identify these mutations by pyrosequencing of pooled *T. circumcincta* larvae collected before and after BZ treatment, but without success [12, 13]. Our hypothesis was that other molecular mechanisms could be implicated in the development of resistance in *T. circumcincta*. However, in one of our studies, the F200Y polymorphism was reported in *Trichostrongylus* spp. larvae collected from the same flocks where the SNPs were absent in *T. circumcincta* [13]. In the present study, we confirmed by means of pyrosequencing the absence of the three conventional mutations (F200Y, F167Y and E198A) in *T. circumcincta* larvae from Spanish sheep flocks, which were resistant to a BZ treatment. Only one flock showed a frequency of the resistant allele of 11.6% at codon 200 after treatment, contradicting the highly resistant phenotype (FECRT = 7%; Hdd = 93.4%). This frequency could be considered as an invalid value since in some studies it was assumed that frequencies equal to or lower than 10% are technical backgrounds [21]. Our hypothesis is that this background could increase when pooled larvae from different species are analysed.

With the aim to clarify the genotype of resistant *T. circumcincta* larvae, a 371-bp fragment including the three codons of interest was sequenced. We verified the susceptible genotype at codons 200 and 167; however, at codon 198, all samples analysed after treatment showed two new polymorphisms at the first two bases of the codon, C and T, leading to CTA, coding for leucine (L). Although this is the first time that CTA is reported at codon 198, the amino acid leucine had been already described in a few previous studies as TTA. The identification of a substitution of glutamic acid (GAA or GAG) with leucine (TTA) at position 198 in *T. circumcincta* on four out of seven farms from the UK was firstly described by Redman et al. [22]. In that study the E198L polymorphism was present with a low frequency in three farms (2.0–8.8%) but with a very high frequency on the remaining one (91.7%). After this initial report, the study was completed with a larger number of farms from the same country (*n* = 164), finding the polymorphism E198L (GAA > TTA) in *T. circumcincta* larvae collected on the majority of farms but with a very low frequency (6.41% in ewes and 7.80% in lambs); only a few of them showed high frequencies, the highest value being 68.76% [23]. However, in these two previous studies, the presence of the E198L polymorphism was not associated with the response to BZ treatment. In Ireland, this new polymorphism was reported with a low frequency (17%), jointly with the SNP F200Y (with a low-medium frequency of 33%), in *T. circumcincta* adults collected after BZ treatment; only one adult nematode collected after treatment carried the polymorphism E198L and was homozygous for the BZ susceptible allele at position 200 (TTC) [24]. E198L (GAA > TTA) was also described in a resistant *T. circumcincta* population with a frequency of 28.1% [25] and also in the same species collected from northern Belgian flocks [26].

There are two other GIN species infecting ruminants in which the polymorphism E198L has been described. In the study carried out by Avramenko et al. [23] and mentioned above, the E198L (GAA > TTA) polymorphism was also detected in *Trichostrongylus axei* at relatively high frequencies on some farms, although the overall mean was 9.90% and 7.09% for ewe and lamb farms, respectively. In *H. contortus*, the presence of leucine (TTA) at codon 198 was present in 82% of all tested samples (*n* = 133) and in 100% of the L3 and pooled adult samples collected after albendazole treatment in goat farms from Sudan [27]. In these samples, the presence of the other SNPs was absent (F167Y, E198A and F200Y). The results of the latter study are in accordance with our findings since in both cases the leucine at codon 198 was the only polymorphism selected after a BZ treatment. Therefore, in both species, *T. circumcincta* and *H. contortus*, it has been confirmed that E198L can confer BZ resistance independently of F200Y or F167Y.

It is noteworthy that in five out of the six studies describing the presence of E198L in three different GIN species, the codon for leucine was TTA (with the exception of the Claerebout et al. study [26], which did not report the codon sequence), rather than CTA as it has been reported in our study. The presence of codon CTA at position 198 was confirmed by sequencing a 371-bp fragment of the β-tubulin gene in different samples, and was particularly evident in all samples collected after treatment. Therefore, the amino acid substitution of glutamic acid (E) with leucine (L) is produced by two consecutive polymorphisms, GAA > CTA or TTA, in both cases, the presence of T at the second position is constant. According to the results obtained after sequencing samples collected before and after treatment (Figs. 2, 3), we designed a pyrosequencing assay to measure the frequencies of G and A, or C and T, independently. With the aim to clarify the amino acid present at codon 198, especially in samples collected before treatment, we supposed that the frequency of G and A, C and T, G and T or C and A should be the same to code for glutamic acid (E), leucine (L), valine (V) or glutamine (Q), respectively. This assumption was supported by the 15 samples analysed after treatment, in all of them the C and T frequencies were very similar, but also in most of the samples collected before treatment (35/39). In fact, a very high correlation was reported between C and T frequencies in the samples collected before treatment (*r* = 0.929; *P* < 0.0001). Consequently, the mean frequency of C and T was calculated to determine the frequency of the polymorphism E198L. However, there are four samples collected before treatment that did not follow this pattern, the frequencies were more similar between G and T, or C and A, coding for valine (GTA) or glutamine (CAA), respectively. Unfortunately, we were not able to distinguish between these genotypes in pooled L1 using a pyrosequencing assay; however, this differentiation was possible using a deep amplicon assay designed by Avramenko et al. [23]. These authors identified an E198V (GAA > GTA) genotype present in almost all the populations in which the E198L (GAA > TTA) occurred. According to Avramenko et al. [23], the codon GTA (V) could be an intermediate step between GAA (E) and TTA (L), or CTA (L) in our case. Taking into account that E198V (GAA > GTA) polymorphism has also been associated with BZ resistance in fungi [28], Avramenko et al. [23] hypothesized that the presence of V could confer an intermediate level of resistance, lower than that of E198L. It is important to mention that the amino acid valine is coded by GTA, with T at the second position of the codon, in the same way as leucine (CTA or TTA). Interestingly, according to our multivariate linear regression Models 1 and 2, the T frequency was the most significant genotypic variable influencing the phenotypic resistance, measured by the FECRT or EHT (*P* < 0.0001). This could open the possibility that the presence of valine (GTA) at codon 198 could be related to BZ resistance as well, although to a lesser extent, since after treatment all genotypes were CTA (L), by sequencing and pyrosequencing. However, as previously mentioned, the presence of other genotypes with low frequencies is difficult to distinguish using either technique.

Although Martin et al. [29] suggested that FECRT and EHT do not detect a resistant phenotype when less than 25% of population carry the resistant genotype, Coles et al. [30] reported that the EHT could be much more sensitive, detecting down to 2–3% of resistant eggs, when a DD is used. In the present study, very high and similar correlations were reported between the genotypic variables (C frequency, T frequency or mean of both frequencies) and phenotypic resistance (*r* > 0.720; *P* < 0.0001), although negatively associated with the FECRT and positively with the Hdd. However, according to the multivariate linear regression analysis, in the EHT 67.1% of the phenotypic variability is associated with the T frequency (model 2) but in the FECRT only 33.4% (model 1). It is important to mention that in the EHT all non-*T. circumcincta* eggs would have failed to hatch reducing the Hdd value and consequently its association with the T frequency. The lower accuracy for detecting the AR by FECRT could be due to the fact that there is a variation in FEC method used to calculate the FECRT, a McMaster technique with a sensitivity of 15. Using a FEC method as FLOTAC, with an analytical sensitivity of 1 epg could have improved the accuracy of the FECRT [31]. Therefore, the EHT using a DD of 0.1 μg/ml of TBZ seems to detect the genotypic resistance determined by E198L more accurately than the FECRT.

## Conclusions

In the present study we have shown that a mutation of glutamic acid (GAA) to leucine (CTA) at codon 198 (E198L) of the isotype-1 β-tubulin gene can confer BZ resistance on its own in *T. circumcincta*. The leucine frequency (L), measured as the mean C and T frequencies (first two bases), was measured in 39 sheep flocks ranging between 10.4 and 80.7% before treatment, and from 82.3 to 92.8% after treatment. Also, high and similar correlations were reported between the genotypic variables (C frequency, T frequency or mean of both frequencies) and phenotypic resistance (*r* > 0.720, *P* < 0.0001), although negatively associated with the FECRT and positively with the EHT. In the EHT, 67.1% of the phenotypic variability is associated with the T frequency (second base) but in the FECRT only 33.4%; therefore, the EHT using a DD seems to detect the genotypic resistance more accurately than the FECRT.

## Supplementary information

**Additional file 1: Table S1.** Individual faecal egg count (FEC) before and after treatment per flock.

**Additional file 2: Table S2.** Faecal egg count (FEC) mean per flock before and after treatment and results of the faecal egg count reduction test (FECRT) (%) and egg hatch test (EHT) (%). Results before treatment: pyrosequencing (% of each base and mean C and T), proportion of *T. circumcincta* carrying the leucine (LEU) amino acid at codon 198 (%) and species identification (%).

**Additional file 3: Table S3.** Results after treatment: pyrosequencing (% of each base and mean C and T), proportion of *T. circumcincta* carrying the leucine (LEU) amino acid at codon 198 (%) and species identification (%).

## Data Availability

All data generated or analysed during this study are included in this published article, and its additional files. The genomic sequences have been deposited in the GenBank database under the accession numbers MT818234, MT818235 and MT818236.

## Abbreviations

AR: Anthelmintic resistance
BZ: Benzimidazole
DD: Discriminant dose
DMSO: Dimethyl sulfoxide
EHT: Egg hatch test
Epg: Eggs per gram
FEC: Faecal egg count
FECRT: Faecal egg count reduction test
GIN: Gastrointestinal nematodes
Hdd: Hatching ration at a discriminant dose
L1: First-stage larvae
MT-PCR: Multiplexed tandem PCR
SNP: Single nucleotide polymorphism
TBZ: Thiabendazole
